# Effects of Naringin on Postharvest Storage Quality of Bean Sprouts

**DOI:** 10.3390/foods11152294

**Published:** 2022-08-01

**Authors:** Xufeng Yang, Yihan Zhao, Qiuming Gu, Weiling Chen, Xinbo Guo

**Affiliations:** 1 College of Food Science, South China Agricultural University, Guangzhou 510642, China; yangxufeng@stu.scau.edu.cn; 2 School of Food Science and Engineering, South China University of Technology, Guangzhou 510640, China; zhaoyihan19980515@163.com; 3 Southern Golden Pomelo Research Institute of Meizhou, Guangdong Lijinyou Agricultural Technology Co., Ltd., Meizhou 514743, China; guqm@163.com (Q.G.); chenweiling1011@163.com (W.C.)

**Keywords:** sprouts, naringin, browning, active substance, postharvest preservation

## Abstract

This study investigated the effects of naringin on soybean and mung bean sprouts postharvest quality. It was found that naringin could maintain the appearance and quality of soybean sprouts and mung bean sprouts during a 6-day storage period as well as delay the occurrence of browning in mung bean sprouts and soybean sprouts. The optimal application rate of naringin was 50–100 μg/mL, which could effectively inhibit the activity of polyphenol oxidase (PPO) and peroxidase (POD) in bean sprouts and increase the ascorbic acid content, where this inhibition response to the browning of mung bean sprouts and soybean sprouts was significantly reduced. Naringin treatment increased gallic acid and p-coumaric acid content in mung bean sprouts as well as the daidzin and rutin content in soybean sprouts, which was also reflected in the improvement of antioxidant activity. The binding of naringin with PPO and POD was analyzed with molecular docking, naringin, and PPO had a lower binding energy (−1.09 Kcal/mol). In conclusion, naringin application in postharvest preservation of mung bean sprouts and soybean sprouts can maintain favorable consumer quality.

## 1. Introduction

Sprouted vegetables are highly nutritious and tender. They are excellent sources of vitamins, dietary fiber, trace minerals, and flavonoids as health foods [1,2]. Sprouts with high moisture content (>95%) and fragile structures are prone to decay and browning during storage. In particular, the hypocotyl of sprouts is easy to turn dark brown. The browning of sprouts will not only affect their appearance, but will also result in the loss of bioactive substances, a reduction in the consumer willingness to buy, and significant financial losses.

Browning is classified into two types: enzymatic browning and non-enzymatic browning. Enzymatic browning is the generation of brown substances from polyphenolic substrates in mung bean sprouts and soybean sprouts, which is catalyzed by major enzymatic browning enzymes such as polyphenol oxidase (PPO) and peroxidase (POD) [2]. Non-enzymatic browning is dominated by the oxidative browning of vitamin C [3]. At present, there are two types of anti-browning solutions for fresh vegetables such as bean sprouts: chemical control and physical prevention [4]. Sikora et al. used ascorbic acid and herbal extracts for chemical control [3], whereas Xiang et al. investigated the influence of plasma-activated water on the physicochemical parameters of mung bean sprouts [5]. There was also the use of ethanol vapor [6], clove essential oil and eugenol [7], phytoncide treatment [4], 4-methoxy cinnamic acid [8], etc. In terms of physical methods, Zhang et al. employed a combination of vacuum and low-temperature technologies to prevent the browning of mung bean sprouts [9], and Gui et al. demonstrated that UV-B irradiation could improve the postharvest quality of mung bean sprouts [10]. Ultrasonic treatment has also been applied to improve the browning of fresh vegetables [11]. However, most of the current methods have problems such as high material costs, significant equipment investments, and toxicological safety. As a result, a cost-effective and safe method to prevent browning should be developed. 

Naringin (40-5,7-trihydroxyflavonone-7-rhamnoglucoside) is a glycoside analog of naringenin, which exhibits a certain bitter taste due to the presence of glycosides. Naringin is abundant in citrus fruit peel, especially grapefruit and pomelo [12,13]. Naringin can also be used as a bittering ingredient in food [14] and is a legal food additive. Previous research has shown that naringin can exert positive effects in reducing lipids [15], antibacterial, anti-inflammatory, and anticancer [16], with health promoting effects. The antioxidant effect of naringin has been extensively studied and confirmed, showing excellent biological activity in both in vivo and in vitro experiments [17,18]. We hypothesized that naringin could also play a good role in delaying the browning of sprouts. Mung bean sprouts and soybean sprouts are the two most widely consumed sprouts in China. Therefore, we sprayed different concentrations of naringin solution on the mung bean sprouts and soybean sprouts and stored them in an incubator at 4 °C. By measuring their physiological and biochemical indices, this study was proposed to find out the optimal concentration of naringin application with the expectation to provide instructions for the application of naringin for the postharvest preservation of soybean sprouts and mung bean sprouts.

## 2. Materials and Methods

### 2.1. Plant Materials

Fresh soybean sprouts and mung bean sprouts were purchased the same day from the local supermarket. Sprouts with intact shape and no breakage damage were selected for subsequent experiments. Naringin powder (purchased from Lijinyou Agricultural Technology Co., Meizhou City, China, purity > 98%) was dissolved and metered to a final concentration of 200 μg/mL with ultrapure water, then the stocking solution was diluted to a different concentration for use. A total of 10 mL of naringin solution was sprayed with different concentrations (25 μg/mL, 50 μg/mL, 100 μg/mL, 150 μg/mL, 200 μg/mL) over the soybean and mung bean sprouts, divided into six portions of 100 g each; the control group was sprayed with 10 mL ultrapure water. The naringin treatment concentrations and the corresponding abbreviations for the sprouts are shown in Table 1. They were placed in polyethylene plastic boxes after treatment and stored in a 4 °C constant temperature incubator. Samples were taken after three and six days of storage, and stored at −40 °C for subsequent analysis of the active substance content, enzymatic activity, and other physicochemical properties.

### 2.2. Vitamin C Analysis

The vitamin C content was determined by the high performance liquid chromatography (HPLC) method [19] with modification. Briefly, the ground sample was added to 0.1% oxalic acid solution, then extracted ultrasonically for 30 min. The mixture was then centrifuged at 9390× *g* for 10 min at 4 °C. The supernatant was collected and added to 25 mM of dithiothreitol vinegar (DTT) solution and 160 mM of buffer, keeping the reaction at room temperature and away from light. The supernatant was filtrated for HPLC detection at the wavelength 245 nm. Vitamin C was separated at 30 °C using the elution phase, which consisted of 5% methanol (*v*/*v*) and 95% 0.1 percent oxalic acid solution (m:V) at a flow rate of 1.0 mL/min. The standard was L- ascorbic acid, and the data are expressed as milligram per gram of fresh weight (μg/g FW).

### 2.3. Polyphenol Composition Analysis

The samples stored at −40 °C were taken out and quickly ground into powder by adding liquid nitrogen. A total of 0.8 g of powder was weighed, put it into a centrifugal tube, added to 1 mL of 80% methanol solution, mixed thoroughly, and ultrasound for 30 min to extract the polyphenols. Samples were centrifuged at 13,523× *g* for 10 min at 25 °C to extract the supernatant. The composition of polyphenols was analyzed by HPLC. The conditions of HPLC were improved according to the methods previously reported in our laboratory [20]. The HPLC analytical conditions were as follows: The detector used was a UV-PDA detector (Waters Company, Milford, MA, USA), C18 column (Waters Company, Milford, MA, USA), column temperature 40 °C, mobile phase 1 mL/min flow rate for the gradient elution, set at three absorption wavelengths of 254 nm, 280 nm, and 320 nm for the determination. The mobile phases were solution A (ultrapure water containing 0.1% TFA) and solution B (acetonitrile containing 0.1% TFA). The elution gradient program was: 0–8 min, 97–95% A; 8–15 min, 95–90% A; 15–25 min, 90–80%; 25–30 min, 80–70%; 30–33 min, 70–65%; 33–52 min, 65–20%; 52–60 min, 20–95%. The polyphenol content of each component was expressed as milligrams per gram of fresh weight (μg/g FW) ± SD.

### 2.4. Polyphenol Oxidase (PPO) and Peroxidase (POD) Analysis

The extraction of the crude enzyme was referred to the reported method [21], 2 g of mung bean sprouts and soybean sprouts were extracted by 20 mL of precooled phosphate buffer [50 mM, pH 7.8, containing 1% polyvinyl pyrrolidone (PVP), and 1 mM ethylene diamine tetra-acetic acid (EDTA)]. The mixture was crushed into a homogenate in an ice bath and then centrifuged at 9390× *g* at 4 °C. The supernatant obtained was the crude enzyme solution, and the solution was stored at 4 °C for analysis.

The analysis of PPO was improved according to the previously reported method [8]. A total of 780 μL of 50 mM, pH 7.8 phosphate buffer, 200 μL of 0.1 M catechol, and 200 μL of crude enzyme extract was added to the centrifuge tube, mixed evenly, and reacted in a water bath at 37 °C. The change in absorbance of the reaction system was measured at 420 nm. The activity of PPO is expressed as U, U = ΔOD_420_/0.0001 min.

The POD analysis was based on the method of Yong-Hua Hu et al. with modification [8]. The crude enzyme extract for 50 μL was added to 150 μL of the POD reaction solution [containing 60% 50 mM phosphate buffer (pH 7.0), 20% 2% hydrogen peroxide solution (*v*/*v*), 20% 1% guaiacol solution (*v*/*v*)]. The change in the absorbance at 470 nm within 60 s was measured rapidly. The activity of POD was expressed as U, U = ΔOD_470_/0.001 min.

### 2.5. Oxygen Radical Absorbance Capacity (ORAC) Analysis

The ORAC analysis was performed according to the method previously reported by our laboratory with modifications [22]. Briefly, 20 μL of sprout extract or Trolox standards was added to each well in the 96-well plate. Incubated at 37 °C for 10 min, 200 μL of the fluorescein sodium solution was added rapidly. Incubated again at 37 °C for 20 min, it was then added to 20 μL of 119.47 mM ABAP solution. The reaction was cycled 35 times in 4.5 min at 485 nm excitation and 535 nm emission using the FilterMax F5 Multi-Mode Microplate Reader (Molecular Devices, San Jose, CA, USA). The ORAC value was expressed in µmol of Trolox equivalents (TE) per gram of fresh weight sample (μmol TE g^−1^ FW).

### 2.6. Polyphenol Oxidase (PPO) 3D Structure Analysis and Molecular Docking

The amino acid sequencing sequence of PPO was obtained from NCBI (www.ncbi.nlm.nih.gov) (accessed on 10 April 2022) and the homologous sequence modeling (SWISS-MODEL, https://swissmodel.expasy.org/) (accessed on 10 April 2022) using mass spectrometry data (serial number: LC100016.1) was a target to predict the 3D structure of PPO. Atodock4 (Scripps Research, San Diego, CA, USA) was used to analyze the molecular docking of PPO, POD and naringin. The crystal structure of POD (PDB code: 1FHF) was obtained from the RCSB PDB protein database. The 3D structure of naringin (Compound CID: 442428) was obtained from the NCBI PubChem database. The conformation with the lowest binding energy was selected as the best docking result [23,24]. The open-source version of PyMOL software (Delano Scientific LLC, Berkeley, CA, USA) was used for visual analysis of the docking results [25].

### 2.7. Statistical Analysis

All results were expressed as the mean ± standard deviation (SD) of triplicates and were analyzed by the IBM SPSS statistical software 26.0 (SPSS, Inc., Chicago, IL, USA). One-way analysis of variance (ANOVA), followed by Duncan’s multiple comparison post-test (*p* < 0.05), was used to compare the differences among groups. Pearson’s correlation coefficient was used to measure the correlation coefficient. 

## 3. Results

The characterization of soybean sprouts and mung bean sprouts sprayed with different concentrations of naringin after three and six days of storage at 4 °C are shown in Supplementary Figure S1. By observing the hypocotyls of the bean sprouts, it could be found that the mung bean sprouts of the control group already showed more obvious browning and wilting after being stored at 4 °C for 3 days. Mung bean sprouts sprayed with 50 g/mL naringin had a similar hypocotyl color to fresh mung bean sprouts, and no significant browning was observed. After 6 days of storage, the mung bean sprouts treated with a 100 μg/mL concentration of naringin had a better appearance, while the browning of mung bean sprouts sprayed with ultrapure water had become severe, with water loss and softness. A similar phenomenon was presented in the soybean sprout experiment.

### 3.1. Effect on Vitamin C Content

**Figure 1:**
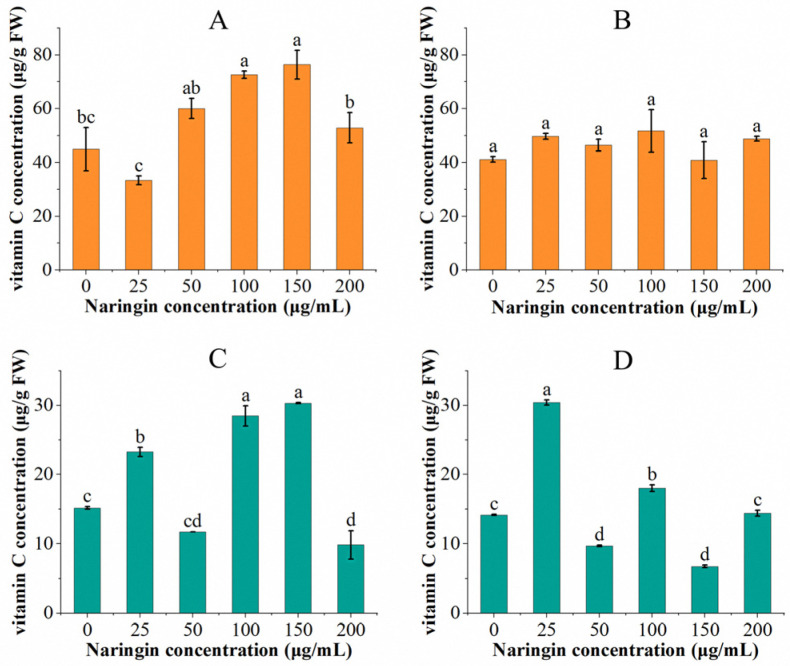
The vitamin C content of soybean sprouts and mung bean sprouts was stored with different concentrations of naringin treatment for 3 and 6 days, respectively. (**A**) Vitamin C content of soybean sprouts after 3 days of storage. (**B**) Vitamin C content of soybean sprouts after 6 days of storage. (**C**) Vitamin C content of mung bean sprouts after 3 days of storage. (**D**) Vitamin C content of mung bean sprouts after 6 days of storage. Bars with different letters indicate significant differences among the samples (*p* < 0.05).

The vitamin C content of soybean sprouts and mung bean sprouts sprayed with different concentrations of naringin and stored for 3 and 6 days are shown in Figure 1. After three days of storage in mung bean sprouts, the vitamin C content of the group treated with 25 μg/mL naringin (23.26 μg/g FW) was significantly higher than that of the control group (15.35 μg/g FW), displayed as 1.5 times. The vitamin C content decreased with increasing naringin concentration, then increased and then decreased, with the highest content occurring in the 150 μg/mL concentration of the naringin treatment group (30.24 μg/g FW) compared to the control group with a double increase. The vitamin C content decreased in all experimental groups after six days of storage, except for the group treated with 25 μg/mL naringin solution, where the vitamin C content (30.77 μg/g FW) increased. The overall trend in vitamin C content was similar to that of the 3 day storage; the lowest content was in the 150 μg/mL concentration naringin treated group (6.79 μg/g FW).

After three days of storage, the vitamin C content in soybean sprouts increased and then decreased as the concentration of naringin treatment increased. The vitamin C content of the 25 μg/mL naringin solution treated group (33.35 μg/g FW) showed a decrease compared to the control group, whereas the 150 μg/mL naringin treated group showed a 0.70-fold increase in the vitamin C content (76.31 μg/g FW) compared to the control group. Similar to the mung bean sprouts, the vitamin C content of soybean sprouts decreased after 6 days of storage in all experimental groups compared to the third day, except for the 25 μg/mL naringin-treated group (49.59 μg/g FW), which showed an increased vitamin C content. The group treated with 100 μg/mL naringin had the highest content of vitamin C (51.66 μg/g FW). Except for the 150 μg/mL naringin-treated group, where the vitamin C content was slightly lower than the control group; all of the experimental groups were higher than the control group.

### 3.2. Effect on Polyphenol Composition

The changes in the polyphenol composition of mung bean sprouts sprayed with different concentrations of naringin solution after 3 and 6 days of storage are shown in Table 2. Four polyphenolic fractions including gallic acid, caffeic acid, p-coumaric acid, and ferulic acid were detected in mung bean sprouts by HPLC analysis, all of which are phenolic acids. After 3 days of storage, the gallic acid, chlorogenic acid, and p-coumaric acid contents of the mung bean sprouts in the naringin treated group were increased, and the content of caffeic acid and p-coumaric acid in the 200 μg/mL naringin treated group was increased by 40% and 26%, compared to the control group, respectively. The content of ferulic acid showed an overall decreasing trend with the increase in naringin concentration. After six days of storage, the control group showed an increase in gallic acid and p-coumaric acid, while the contents of caffeic acid and ferulic acid decreased. The content of gallic acid in the treated group was reduced overall than on the third day. The content of caffeic acid increased in all treatment groups except for a significant decrease in the 200 μg/mL naringin treated group. p-coumaric acid followed the same pattern as caffeic acid.

The changes in the polyphenol composition of soybean bean sprouts sprayed with different concentrations of naringin solution after 3 and 6 days of storage are shown in Table 3. Gallic acid, daidzin, rutin, and genistein were found as polyphenolic components in soybean sprouts, predominantly as flavonoids. The change in gallic acid after three days of storage was not significant. The content of daidzin showed an overall increasing trend with naringin concentration with the highest in the 200 μg/mL naringin treated group (26.06 μg/g FW). The content of both rutin and genistein was higher in the treated group than in the control group, with the rutin content being highest in the 50 μg/mL naringin-treated group (35.88 μg/g FW) and the genistein content being highest in the 200 μg/mL concentration naringin-treated group (77.16 μg/g FW). After 6 days of storage, the content of gallic acid increased, and the content of daidzin decreased in the control, 25 μg/mL, and 200 μg/mL naringin treatment groups while increasing in the remaining treatment groups. The content of rutin was slightly decreased in the control group but increased in all of the treated groups, with the same pattern as at 3 days. All of the genistein content increased relative to three days.

### 3.3. Effect on Polyphenol Oxidase (PPO) and Peroxidase (POD) Activity

**Figure 2:**
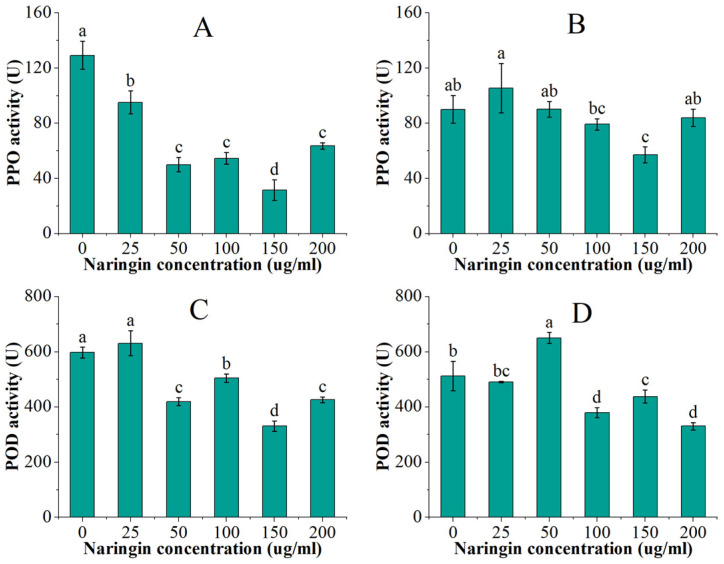
The PPO and POD activities of the mung bean sprouts. (**A**) PPO activity after 3 days of storage; (**B**) PPO activity after 6 days of storage; (**C**) POD activity after 3 days of storage; (**D**) POD activity after 6 days of storage. Bars with different letters indicate significant differences among the samples (*p* < 0.05).

PPO and POD are the two most important enzymes associated with enzymatic browning. The PPO and POD activities of mung bean sprouts are shown in Figure 2. After 3 days of storage, PPO activity in mung bean sprouts showed a trend of decreasing and then increasing with naringin concentration, which were all significantly lower compared to the control group (*p* < 0.5). The lowest activity was found in the 150 μg/mL naringin-treated group, which was 1/4 of the control group activity. The POD activity was significantly lower (*p* < 0.5) in all the groups treated with more than 25 μg/mL naringin, which was slightly higher than the control group. The group treated with 50 μg/mL naringin had the lowest POD activity, which was 44% lower than the control group. The PPO and POD enzyme activities decreased overall after 6 days of storage, and the trend of changes in the groups treated with different concentrations of naringin was similar to that at 3 days, reaching the lowest in the 150 μg/mL and 200 μg/mL treatment groups, respectively.

**Figure 3:**
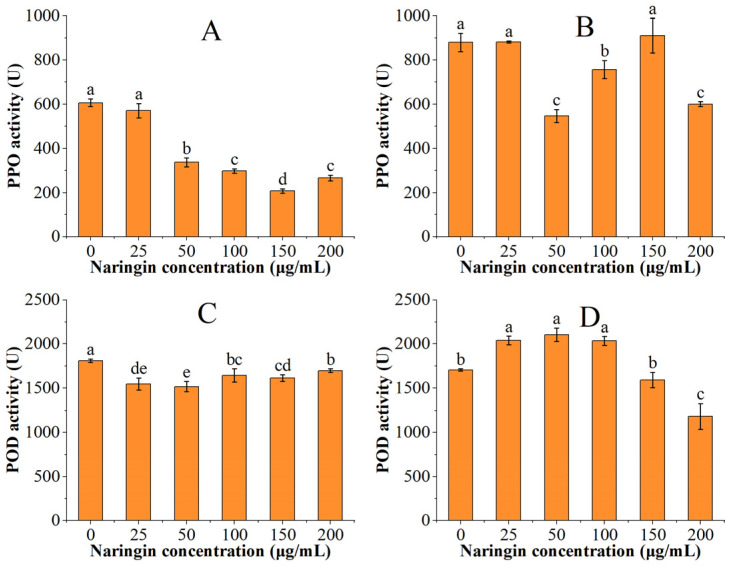
The PPO and POD activities of soybean sprouts. (**A**) PPO activity after 3 days of storage; (**B**) PPO activity after 6 days of storage; (**C**) POD activity after 3 days of storage; (**D**) POD activity after 6 days of storage. Bars with different letters indicate significant differences among the samples (*p* < 0.05).

The PPO and POD activities of the soybean sprouts are shown in Figure 3. The PPO and POD activities were significantly higher in the soybean sprouts than in the mung bean sprouts. The PPO activity decreased with increasing naringin concentration after three days of storage and reached the lowest in the 150 μg/mL naringin-treated group, which was 34% of the control group. The POD activity decreased and then increased, reaching the minimum in the 50 μg/mL naringin treated group. After 6 days of storage, the PPO activity was lowest in the 50 μg/mL naringin-treated group, which was 62% of the control group. The POD activity showed an increasing trend followed by a decreasing trend, reaching the highest in the 50 μg/mL naringin-treated group. This trend was opposite to that of the POD activity of mung bean sprouts, which might be related to the different absorption and utilization of naringin in different species.

### 3.4. Effect on Oxygen Radical Absorbance Capacity

**Figure 4:**
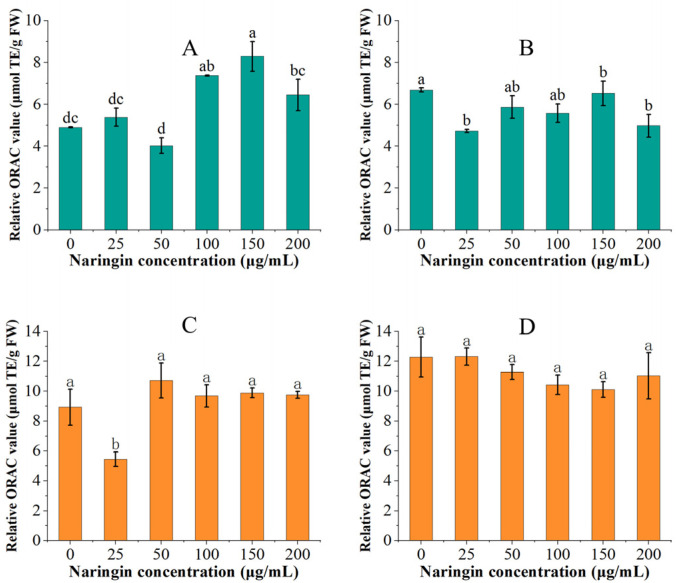
The ORAC values of mung bean sprouts and soybean sprouts, (**A**): ORAC values of mung bean sprouts after 3 days of storage, (**B**): ORAC values of mung bean sprouts after 6 days of storage, (**C**): ORAC values of soybean sprouts after 3 days of storage, (**D**): ORAC values of soybean sprouts after 6 days of storage. Bars with different letters indicate significant differences among the samples (*p* < 0.05).

The variation of oxygen radical absorbance capacity for mung bean sprouts and soybean sprouts is shown in Figure 4. In the ORAC experiment, we discovered that the ORAC values of both mung bean sprouts and soybean sprouts in the control and low concentration (<100 μg/mL) naringin-treated groups increased with the extension of storage time, while the ORAC values of the high concentration (≥100 μg/mL) naringin-treated group decreased. Under the effect of naringin, the ORAC values of the mung bean sprouts stored for 3 days were significantly increased, reaching the highest 1.67 times that of the control group (8.287 μmol TE/g FW). The ORAC value of soybean sprouts stored for three days in the 50 μg/mL naringin-treated group reached 1.2 times that of the control group (10.714 μmol TE/g FW). However, after 6 days of storage, the ORAC values of both mung bean sprouts and soybean sprouts in the naringin-treated group were lower than those of the control group. The ORAC value of soybean sprouts increased relative to three days of storage, whilst the ORAC value of mung bean sprouts decreased relative to three days of storage.

### 3.5. Molecular Docking Result

**Figure 5:**
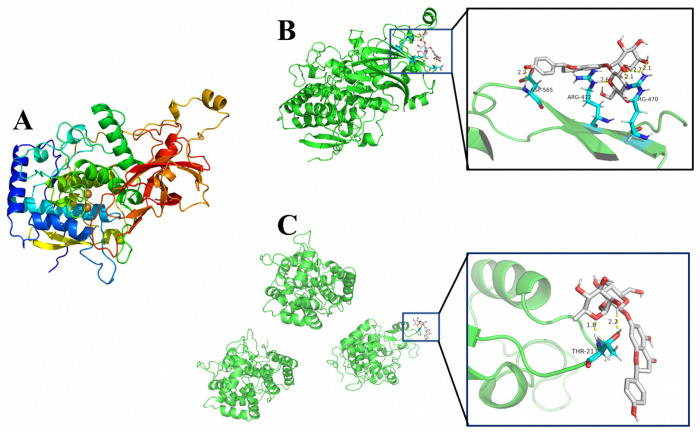
The 3D structure and molecular docking results. (**A**). The 3D crystal structure of PPO, (**B**) PPO and naringin docking results, (**C**) POD and naringin docking results.

The 3D crystal structure of PPO was obtained by homologous sequence construction (SWISS-MODEL, https://swissmodel.expasy.org/) (accessed on 10 April 2022) as shown in Figure 5A. The molecular docking analysis of PPO, POD, and naringin was performed by autodock4 software and the docking results were visualized using the open source version of PyMOL software. The results are shown in Figure 5B,C. The lowest binding energy of PPO and naringin was −1.09 Kcal/mol, forming three hydrogen bonding interactions at the position of residue ARG-470 and one hydrogen bonding interaction at the positions of residues ARG-472 and ASP-565 each. The lowest binding energy of POD and naringin was −0.53 Kcal/mol, and two hydrogen bonding interactions were formed at the THR-232 residues in the A region.

## 4. Discussion

Through the observation of the appearance characterization of bean sprouts, naringin can better prevent browning during the storage of sprouts while reducing the water loss and softening of sprouts, enabling sprouts to maintain the appearance of a more intact sale. PPO and POD are positively correlated with the browning of bean sprouts. Yellow-brown quinones are produced by PPO using polyphenols as substrates [26], while POD employs hydrogen peroxide as an electron acceptor to catalyze the oxidation of the substrate [27]. The PPO activity of both the mung bean sprouts and soybean sprouts was significantly reduced after naringin treatment, and the effect was significant for the samples after three days of storage. It was shown that naringin affected the rate of the PPO catalysis of polyphenolic substances, and a non-competitive inhibition occurred, thus reducing the activity of PPO [28,29]. The oxidative stress formed by high concentrations of naringin (200 μg/mL) prompted an increase in the content of some phenolic acids, which attenuated the non-competitive inhibitory effect of naringin [30,31]. The inhibitory effect of naringin on PPO was worse after six days of storage than after three days; this situation was similar to the phenomenon in the experiment using the *Rosa roxburghii* extract to prevent browning in apple juice by KaiboYu et al., which may be related to the oxidative consumption of naringin [32]. Recent studies have shown that important substrates of PPO are flavonoids [9,33]. The flavonoid content of soybean sprouts was significantly higher than that of mung bean sprouts, which is why the PPO activity of soybean sprouts was significantly higher than that of mung bean sprouts. Naringin was found to have a significant inhibitory effect on the POD activity of mung bean sprouts in the POD activity experiments, but a poor inhibitory effect on the POD activity of soybean sprouts. This indicates that naringin mainly inhibits the enzymatic browning of sprouts by inhibiting the PPO activity.

According to the molecular docking results, naringin and PPO had lower binding energy than naringin and POD. The reaction will be more favorable if the binding energy is lower [24]. The number of hydrogen bonds formed by naringin and PPO was also higher than that of POD. It indicates that naringin is much easier to combine with PPO and has a greater effect on the structure of PPO, thus affecting the binding between PPO and the reaction substrate, reducing the catalytic efficiency of PPO. This conclusion is also consistent with the results of tests on PPO and POD activity.

The non-enzymatic browning of fresh vegetables is dominated by the oxidative decomposition of vitamin C [34]. As an important nutritional supplement, the content of vitamin C is also an important index of the nutritional value of vegetables. Therefore, it is important to maintain the vitamin C content in sprouts. After the application of naringin, both mung bean sprouts and soybean sprouts had higher vitamin C content than the control group, with the highest level in the treated group being twice that of the control group after three days of storage for mung bean sprouts and 1.4 times that of the control group for the treated group of soybean sprouts. This shows that naringin can effectively inhibit the occurrence of non-enzymatic browning in sprouts. Related studies have shown that naringin has excellent antioxidant and free radical scavenging ability, which can effectively reduce the production of free radicals in the organism [35,36]. The use of naringin forms an antioxidant film on the surface of the sprouts, which reacts preferentially with oxygen. Thus, the addition of naringin reduced the free radical attack on vitamin C while increasing the vitamin C content and presenting a certain dose-dependent protective effect. In contrast, a high concentration of naringin (200 μg/mL) formed a stress, leading to vitamin C loss and a diminished protective effect of naringin. It is postulated that the substantial reduction in vitamin C consumption is not only related to the reduction of free radicals, but lower concentrations of naringin can affect the expression of related enzymes in the metabolic pathway of vitamin C such as the inhibition of ascorbic acid oxidase enzyme activity, which slows down the oxidation of vitamin C. Interestingly, we found that the vitamin C content of the lowest concentration naringin treated group (25 μg/mL) had increased after six days of storage. Low-intensity exogenous stimulation can put cells in a state of oxidative stress and induce them to enhance their antioxidant system and synthesize a variety of low-molecular compounds including ascorbic acid [37,38]. The low concentration of naringin (25 μg/mL) allowed the bean sprouts to trigger this natural resistance mechanism, leading to an increase in the vitamin C content during the storage process [25].

Since naringin inhibited the activity of PPO and POD, which affected the reaction between the PPO and substrate to some extent, the content of some polyphenols was increased. Phenolic acids were significantly higher in the group treated with high concentrations of naringin (200 μg/mL), presumably due to oxidative stress in the sprouts. A similar phenomenon was observed by Sikora et al. in experiments on the action of vitamin C on mung bean sprouts [3]. After 6 days of storage, the flavonoid content of soybean sprouts and mung bean sprouts increased. Flavonoid substances are produced downstream of the polyphenol metabolic pathway; as the storage time becomes longer, various phenolic substances enter the downstream reaction, and the accumulation of flavonoid substances increases. Changes in phenolic acids and flavonoids indicate that naringin may impact downstream of the phenylpropane pathway. It inhibited the polyphenol metabolic pathway synthesis toward naringin, while positively regulating the synthesis toward flavones and flavanols. Previous studies have also shown an association between increased antioxidant activity and increased expression of the phenylpropane pathway, allowing for the evaluation of phenylalanine ammonia-lyase activity in future studies.

Earlier studies have shown that phenolic acid compounds are the primary antioxidant substances in mung bean sprouts [39,40], and the content of phenolic acids was increased in both the soybean sprouts and mung bean sprouts in the treated groups, so the ORAC values were also increased. Phenolic acids have a higher oxygen radical absorbance capacity than flavonoids, and the percentage of flavonoids in soybean sprouts was higher, which is why naringin had no significant effect on the ORAC value of the soybean sprouts. Vitamin C is an excellent antioxidant in food systems [41], helping to maintain the active state of many bioactive compounds such as vitamin E, flavonoids, and some phenolic acid. The vitamin C content was significantly increased under the effect of naringin, therefore, the flavonoids maintained better activity and the ORAC value increased [37]. 

## 5. Conclusions

This study found that the treatment effect of a 50–100 μg/mL concentration of naringin was optimal to maintain the favorable consumer quality for soybean sprouts and mung bean sprouts during the storage period of 6 days. Naringin was able to exert a significant effect on the anti-browning of soybean sprouts and mung bean sprouts. It has a significant inhibitory effect on ascorbic acid oxidation and enzymatic browning, can effectively inhibit the activity of PPO and POD, and increase the content of vitamin C. Additionally, it also increased the content of gallic acid, p-coumaric acid, daidzein, and genistein, which improved the antioxidant capacity of bean sprouts at the same time. Further studies on the mechanism of the anti-browning effect of naringin are necessary. Naringin is inexpensive and can be obtained from the by-products of grapefruit cultivation and production. The results of this study provide strong support for the use of naringin as a preservative for soybean sprouts and mung bean sprouts while improving their nutritional quality. The present results also indicate the possibility of promoting naringin as a plant-based preservative for the postharvest treatment of fresh fruits and vegetables.

## Figures and Tables

**Table 1:** The corresponding abbreviations for the soybean sprouts and mung bean sprouts.

Naringin Concentration (μg/mL)	0	25	50	100	150	200
Soybean sprouts treatment abbreviations	S1	S2	S3	S4	S5	S6
Mung bean sprouts treatment abbreviations	M1	M2	M3	M4	M5	M6

**Table 2:** The changes in polyphenol fractions of mung bean sprouts sprayed with different concentrations of naringin after 3 and 6 days of storage, the unit is μg/g FW. Three days: results of treatment after storage for three days; six days: results of treatment after storage for six days. Data (Means ± SD) followed by the different letter in the same line indicate statistically significant difference (*p* < 0.05).

Polyphenols (μg/g FW)	Processing Time	M1	M2	M3	M4	M5	M6
gallic acid	3 d	11.40 ± 0.38 a	14.27 ± 0.53 a	11.70 ± 1.13 a	14.07 ± 2.21 a	12.50 ± 0.76 a	11.95 ± 0.36 a
	6 d	14.12 ± 0.15 a	11.39 ± 0.17 cd	12.42 ± 0.05 bc	12.81 ± 0.36 b	11.08 ± 0.24 d	10.81 ± 0.52 d
caffeic acid	3 d	19.40 ± 0.17 b	17.71 ± 0.11 bc	15.77 ± 0.14 cd	16.93 ± 0.70 cd	15.26 ± 0.04 d	24.53 ± 1.35 a
	6 d	16.09 ± 0.16 a	17.47 ± 2.45 a	18.81 ± 0.19 a	18.45 ± 2.01 a	18.03 ± 1.97 a	14.07 ± 5.47 a
p-coumaric acid	3 d	20.88 ± 0.64 c	25.14 ± 0.53 abc	26.42 ± 1.45 bc	24.27 ± 0.65 bc	24.84 ± 1.46 bc	29.36 ± 1.86 a
	6 d	31.76 ± 0.04 a	23.93 ± 0.05 d	25.55 ± 0.18 cd	32.24 ± 2.34 a	27.80 ± 0.79 bc	30.78 ± 0.03 ab
ferulic acid	3 d	5.43 ± 1.58 a	4.63 ± 1.12 a	3.52 ± 0.10 a	4.12 ± 0.47 a	3.88 ± 0.09 a	3.53 ± 0.20 a
	6 d	3.52 ± 0.05 ab	3.55 ± 0.20 ab	3.71 ± 0.20 a	3.67 ± 0.34 a	2.96 ± 0.06 b	4.01 ± 0.03 a

**Table 3:** The changes in the polyphenol fractions of soybean sprouts sprayed with different concentrations of naringin after 3 and 6 days of storage, the unit is μg/g FW. Three days: results of treatment after storage for three days; six days: results of treatment after storage for six days. Data (Means ± SD) followed by the different letter in the same line indicate statistically significant difference (*p* < 0.05).

Polyphenols (μg/g FW)	Processing Time	S1	S2	S3	S4	S5	S6
gallic acid	3 d	19.29 ± 0.09 a	18.06 ± 0.36 ab	18.00 ± 0.01 ab	19.25 ± 0.62 a	17.77 ± 0.55 b	19.13 ± 0.11 ab
	6 d	21.54 ± 0.51 a	20.27 ± 0.19 a	21.78 ± 0.08 a	20.20 ± 1.31 a	19.72 ± 2.06 a	19.74 ± 0.01 a
daidzin	3 d	17.07 ± 1.15 b	19.49 ± 2.17 ab	17.91 ± 1.28 ab	19.74 ± 1.83 ab	22.80 ± 2.72 ab	26.06 ± 1.30 a
	6 d	16.28 ± 2.64 a	17.38 ± 1.39 a	18.48 ± 0.71 a	20.46 ± 1.38 a	23.67 ± 2.50 a	23.48 ± 3.06 a
rutin	3 d	27.44 ± 1.44 a	31.82 ± 2.35 a	35.88 ± 2.39 a	30.00 ± 2.58 a	35.01 ± 4.79 a	33.63 ± 0.56 a
	6 d	26.50 ± 5.24 a	27.95 ± 3.95 a	32.28 ± 5.99 a	30.54 ± 2.45 a	36.12 ± 4.69 a	34.13 ± 5.03 a
genistein	3 d	55.85 ± 3.46 b	61.49 ± 4.42 b	65.41 ± 2.13 ab	63.88 ± 5.00 ab	60.78 ± 2.29 b	77.16 ± 6.18 a
	6 d	56.68 ± 0.26 b	69.25 ± 0.66 ab	64.73 ± 1.45 b	66.24 ± 6.61 b	67.92 ± 7.95 ab	84.70 ± 4.35 a

## Data Availability

The date are available from the corresponding author.

## Supplementary Materials

The following supporting information can be downloaded at: https://www.mdpi.com/article/10.3390/foods11152294/s1, Figure S1: The characterization of soybean sprouts and mung bean sprouts sprayed with different concentrations of naringin after three and six days of storage at 4 °C.

## References

[1] Ha M.C., Im D.Y., Park H.S., Dhungana S.K., Kim I.D., Shin D.H. (2022). Seed Treatment with Illite Enhanced Yield and Nutritional Value of Soybean Sprouts. Molecules.

[2] Lv C.Y., Zhao G.H., Ning Y. (2017). Interactions between plant proteins/enzymes and other food components, and their effects on food quality. Crit. Rev. Food Sci. Nutr..

[3] Sikora M., Swieca M. (2018). Effect of ascorbic acid postharvest treatment on enzymatic browning, phenolics and antioxidant capacity of stored mung bean sprouts. Food Chem..

[4] Kim D.H., Kim H.B., Chung H.S., Moon K.D. (2014). Browning control of fresh-cut lettuce by phytoncide treatment. Food Chem..

[5] Xiang Q., Liu X., Liu S., Ma Y., Xu C., Bai Y. (2019). Effect of plasma-activated water on microbial quality and physicochemical characteristics of mung bean sprouts. Innov. Food Sci. Emerg. Technol..

[6] Goyal A., Siddiqui S. (2014). Effects of ultraviolet irradiation, pulsed electric field, hot water dip and ethanol vapours treatment on keeping and sensory quality of mung bean (*Vigna radiata* L. Wilczek) sprouts. J. Food Sci. Technol..

[7] Chen X., Ren L., Li M., Qian J., Fan J., Du B. (2017). Effects of clove essential oil and eugenol on quality and browning control of fresh-cut lettuce. Food Chem..

[8] Hu Y.-H., Chen C.-M., Xu L., Cui Y., Yu X.-Y., Gao H.-J., Wang Q., Liu K., Shi Y., Chen Q.-X. (2015). Postharvest application of 4-methoxy cinnamic acid for extending the shelf life of mushroom (*Agaricus bisporus*). Postharvest Biol. Technol..

[9] Zhang S.J., Hu T.T., Liu H.K., Chen Y.Y., Pang X., Zheng L., Chang S., Kang Y. (2018). Moderate vacuum packing and low temperature effects on qualities of harvested mung bean (*Vigna radiata* L.) sprouts. Postharvest Biol. Technol..

[10] Gui M., He H., Li Y., Chen X., Wang H., Wang T., Li J. (2018). Effect of UV-B treatment during the growth process on the postharvest quality of mung bean sprouts (*Vigna radiata*). Int. J. Food Sci. Technol..

[11] Pan Y., Chen L., Pang L., Chen X., Jia X., Li X. (2020). Ultrasound treatment inhibits browning and improves antioxidant capacity of fresh-cut sweet potato during cold storage. RSC Adv..

[12] Igual M., García-Martínez E., Camacho M.M., Martínez-Navarrete N. (2013). Jam processing and storage effects on β-carotene and flavonoids content in grapefruit. J. Funct. Foods.

[13] Yusof S., Ghazali H.M., King G.S. (1990). Naringin content in local citrus fruits. Food Chem..

[14] Li X.R., Liu H.Y., Wu X.Z., Xu R.N., Ma X.Y., Zhang C.X., Song Z.Z., Peng Y.R., Ni T.J., Xu Y.T. (2021). Exploring the interactions of naringenin and naringin with trypsin and pepsin: Experimental and computational modeling approaches. Spectrochim. Acta Part A-Mol. Biomol. Spectrosc..

[15] Zhang X., Zhang Y., Gao W., Guo Z., Wang K., Liu S., Duan Z., Chen Y. (2021). Naringin improves lipid metabolism in a tissue-engineered liver model of NAFLD and the underlying mechanisms. Life Sci..

[16] Chen R., Qi Q.L., Wang M.T., Li Q.Y. (2016). Therapeutic potential of naringin: An overview. Pharm. Biol..

[17] Bacanli M., Basaran A.A., Basaran N. (2015). The antioxidant and antigenotoxic properties of citrus phenolics limonene and naringin. Food Chem. Toxicol..

[18] Cavia-Saiz M., Busto M.D., Pilar-Izquierdo M.C., Ortega N., Perez-Mateos M., Muniz P. (2010). Antioxidant properties, radical scavenging activity and biomolecule protection capacity of flavonoid naringenin and its glycoside naringin: A comparative study. J. Sci. Food Agric..

[19] Liu F., Xiang N., Hu J.G., Shijuan Y., Xie L., Brennan C.S., Huang W., Guo X. (2017). The manipulation of gene expression and the biosynthesis of Vitamin C, E and folate in light-and dark-germination of sweet corn seeds. Sci. Rep..

[20] Lu Y., Chang X., Guo X. (2019). Dynamic Changes of Ascorbic Acid, Phenolics Biosynthesis and Antioxidant Activities in Mung Beans (*Vigna radiata*) until Maturation. Plants.

[21] Xu F., Tang Y., Dong S., Shao X., Wang H., Zheng Y., Yang Z. (2016). Reducing yellowing and enhancing antioxidant capacity of broccoli in storage by sucrose treatment. Postharvest Biol. Technol..

[22] Xiang N., Wen T.X., Yu B.L., Li G.K., Li C.Y., Li W., Lu W.J., Hu J.G., Guo X.B. (2020). Dynamic effects of post-harvest preservation on phytochemical profiles and antioxidant activities in sweet corn kernels. Int. J. Food Sci. Technol..

[23] Liao T., Zhou L., Liu J.P., Zou L.Q., Dai T.T., Liu W. (2021). Inhibitory mechanism of salicylic acid on polyphenol oxidase: A cooperation between acidification and binding effects. Food Chem..

[24] Nokthai P., Lee V.S., Shank L. (2010). Molecular Modeling of Peroxidase and Polyphenol Oxidase: Substrate Specificity and Active Site Comparison. Int. J. Mol. Sci..

[25] Zhou T., Wang P., Gu Z., Ma M., Yang R. (2020). Spermidine improves antioxidant activity and energy metabolism in mung bean sprouts. Food Chem..

[26] Yao F., Huang Z., Li D., Wang H., Xu X., Jiang Y., Qu H. (2014). Phenolic components, antioxidant enzyme activities and anatomic structure of longan fruit pericarp following treatment with adenylate triphosphate. Sci. Hortic..

[27] Lin Y., Lin H., Lin Y., Zhang S., Chen Y., Jiang X. (2016). The roles of metabolism of membrane lipids and phenolics in hydrogen peroxide-induced pericarp browning of harvested longan fruit. Postharvest Biol. Technol..

[28] Holzwarth M., Wittig J., Carle R., Kammerer D.R. (2013). Influence of putative polyphenoloxidase (PPO) inhibitors on strawberry (*Fragaria × ananassa* Duch.) PPO, anthocyanin and color stability of stored purées. LWT Food Sci. Technol..

[29] Sukhonthara S., Kaewka K., Theerakulkait C. (2016). Inhibitory effect of rice bran extracts and its phenolic compounds on polyphenol oxidase activity and browning in potato and apple puree. Food Chem..

[30] Guo L., Ma Y., Shi J., Xue S. (2009). The purification and characterisation of polyphenol oxidase from green bean (*Phaseolus vulgaris* L.). Food Chem..

[31] Sikora M., Świeca M., Franczyk M., Jakubczyk A., Bochnak J., Złotek U. (2019). Biochemical Properties of Polyphenol Oxidases from Ready-to-Eat Lentil (*Lens culinaris* Medik.) Sprouts and Factors Affecting Their Activities: A Search for Potent Tools Limiting Enzymatic Browning. Foods.

[32] Yu K., Zhou L., Sun Y., Zeng Z., Chen H., Liu J., Zou L., Liu W. (2021). Anti-browning effect of Rosa roxburghii on apple juice and identification of polyphenol oxidase inhibitors. Food Chem..

[33] Queiroz C., Mendes Lopes M.L., Fialho E., Valente-Mesquita V.L. (2008). Polyphenol Oxidase: Characteristics and Mechanisms of Browning Control. Food Rev. Int..

[34] Adams J.B., Brown H.M. (2007). Discoloration in Raw and Processed Fruits and Vegetables. Crit. Rev. Food Sci. Nutr..

[35] Diaz-Uribe C.E., Vallejo W., Oliveros G., Muñoz A. (2016). Study of scavenging capacity of naringin extracted from Citrus uranium peel against free radicals. Prospectiva.

[36] Fu H., Lin M., Katsumura Y., Muroya Y. (2011). Free-Radical Scavenging Activities of Silybin and Its Analogues: A Pulse Radiolysis Study. Int. J. Chem. Kinet..

[37] Burguieres E., McCue P., Kwon Y.-I., Shetty K. (2007). Effect of vitamin C and folic acid on seed vigour response and phenolic-linked antioxidant activity. Bioresour. Technol..

[38] Wang H., Gui M., Tian X., Xin X., Wang T., Li J. (2017). Effects of UV-B on vitamin C, phenolics, flavonoids and their related enzyme activities in mung bean sprouts (*Vigna radiata*). Int. J. Food Sci. Technol..

[39] Gan R.-Y., Lui W.-Y., Chan C.-L., Corke H. (2017). Hot Air Drying Induces Browning and Enhances Phenolic Content and Antioxidant Capacity in Mung Bean (*Vigna radiata* L.) Sprouts. J. Food Process. Preserv..

[40] Gan R.-Y., Wang M.-F., Lui W.-Y., Wu K., Corke H. (2016). Dynamic changes in phytochemical composition and antioxidant capacity in green and black mung bean (*Vigna radiata*) sprouts. Int. J. Food Sci. Technol..

[41] Jang J.-H., Moon K.-D. (2011). Inhibition of polyphenol oxidase and peroxidase activities on fresh-cut apple by simultaneous treatment of ultrasound and ascorbic acid. Food Chem..

