# Supplementary material for: Effects of Naringin on Postharvest Storage Quality of Bean Sprouts
Source: Foods. 2022 Aug 1;11(15):2294. doi: 10.3390/foods11152294 (PMC9368302; doi:10.3390/foods11152294)
Supplement: Supplementary file 1 [file foods-11-02294-s001.zip › foods-1808693-Figure S1.pdf]

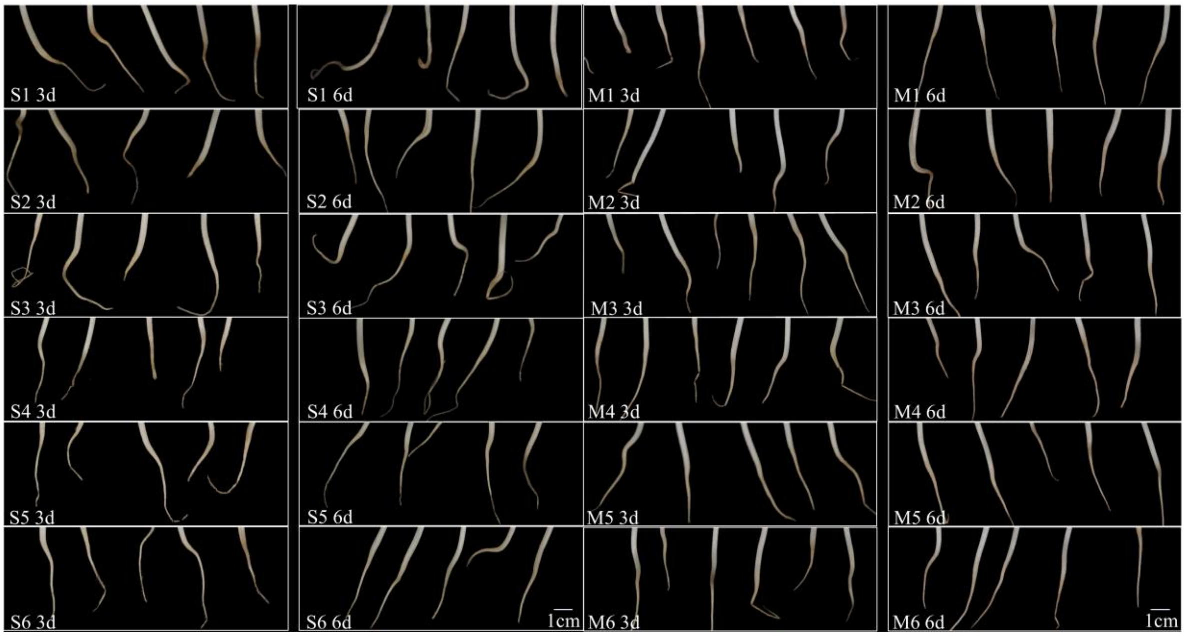

Figure S1. The characterization of soybean sprouts and mung bean sprouts sprayed with different concentrations of naringin after three and six days of storage at 4 °C.
